# Effects of protease and non-starch polysaccharide enzyme on performance, digestive function, activity and gene expression of endogenous enzyme of broilers

**DOI:** 10.1371/journal.pone.0173941

**Published:** 2017-03-21

**Authors:** Lin Yuan, Mingfa Wang, Xiaotu Zhang, Zhixiang Wang

**Affiliations:** 1 College of Animal Science and Veterinary Medicine, Henan Agricultural University, Zhengzhou, China; 2 Institute of Animal Husbandry and Veterinary Medicine, Henan Academy of Agricultural Sciences, Zhengzhou, China; Leibniz-Institut fur Pflanzengenetik und Kulturpflanzenforschung Gatersleben, GERMANY

## Abstract

Three hundred one-day-old male broiler chickens (Ross-308) were fed corn-soybean basal diets containing non-starch polysaccharide (NSP) enzyme and different levels of acid protease from 1 to 42 days of age to investigate the effects of exogenous enzymes on growth performance, digestive function, activity of endogenous digestive enzymes in the pancreas and mRNA expression of pancreatic digestive enzymes. For days 1-42, compared to the control chickens, average daily feed intake (ADFI) and average daily gain (ADG) were significantly enhanced by the addition of NSP enzyme in combination with protease supplementation at 40 or 80 mg/kg (p<0.05). Feed-to-gain ratio (FGR) was significantly improved by supplementation with NSP enzymes or NSP enzyme combined with 40 or 80 mg/kg protease compared to the control diet (p<0.05). Apparent digestibility of crude protein (ADCP) was significantly enhanced by the addition of NSP enzyme or NSP enzyme combined with 40 or 80 mg/kg protease (p<0.05). Cholecystokinin (CCK) level in serum was reduced by 31.39% with NSP enzyme combined with protease supplementation at 160 mg/kg (p<0.05), but the CCK level in serum was increased by 26.51% with NSP enzyme supplementation alone. After 21 days, supplementation with NSP enzyme and NSP enzyme combined with 40 or 80 mg/kg protease increased the activity of pancreatic trypsin by 74.13%, 70.66% and 42.59% (p<0.05), respectively. After 42 days, supplementation with NSP enzyme and NSP enzyme combined with 40 mg/kg protease increased the activity of pancreatic trypsin by 32.45% and 27.41%, respectively (p<0.05). However, supplementation with NSP enzyme and 80 or 160 mg/kg protease decreased the activity of pancreatic trypsin by 10.75% and 25.88%, respectively (p<0.05). The activities of pancreatic lipase and amylase were significantly higher in treated animals than they were in the control group (p<0.05). Supplementation with NSP enzyme, NSP enzyme combined with 40 or 80 mg/kg protease increased pancreatic trypsin mRNA levels by 40%, 44% and 28%, respectively. Supplementation with NSP enzyme and 160 mg/kg protease decreased pancreatic trypsin mRNA levels by 13%. Pancreatic lipase and amylase mRNA expression were significantly elevated in treated animals compared to the control group (p<0.05). These results suggest that the amount of NSP enzyme and acid protease in the diet significantly affects digestive function, endogenous digestive-enzyme activity and mRNA expression in broilers.

## Introduction

Soybean meal (SBM) is an important protein resource in poultry diets due to its high protein content and amino-acid balance. However, the nutritional value of SBM is reduced by the inclusion of non-starch polysaccharides (NSPs) and other anti-nutritional factors (ANFs) that cause poultry meal digestion to be incomplete[1, 2]. There are large variations in the nutrient contents of soybean meal. This difference is not only reflected in the composition of proteins and amino acids, but also in the levels of NSPs and other anti-nutritional factors[3]. Water-soluble NSPs are difficult to digest and decrease the amount of free water in the intestine, thus affecting the digestion and absorption of other nutrients[4].

The addition of enzymes can reduce the negative effects of NSPs and improve nutrient availability in poultry diets. The hydrolysis of NSPs can reduce the stickiness of pentosan, release nutrients from the cell wall, and break down starches into simple sugars, allowing nutrients as well as digestive enzymes to move more freely and improving growth performance, nutrient absorption and the efficiency of feed digestion. Previous trials have demonstrated that proteases and carbohydrate enzymes can improve the nutritional value of SBM[5–8]. Enzymes added to poultry diets can improve weight gain, feed conversion, the viscosity of intestinal chyme, and the digestibility of dry matter [9–11].

The primary benefit of xylanase in feed is to the reduction in viscosity. Partial hydrolysis of NSP by xylanase decreases intestinal chyme viscosity[12] and degrades cell-wall polysaccharides to release nutrients[3, 13]. Gao et al. (2008) speculated that its growth-promoting effect may also be related to the oligosaccharides produced by endogenous enzymes or exogenous enzymes[14].

Due to the inadequate secretion of digestive enzymes by broilers, exogenous enzymes have been included in broiler diets for decades. The supplementation of corn-soybean-meal diets with exogenous enzymes can improve weight gain, feed conversion ratio and ileal digestibility of amino acids[10]. Supplementation improves the availability of calories, protein and other nutrients. Proteases added to soybean meal broiler diets can increase body weight gain, apparent nitrogen retention and apparent metabolizable energy, although the feed-conversion ratio remains unchanged[15].

Exogenous enzymes may also increase the secretion of endogenous substances in the gastrointestinal tract of broilers[16–18]. However, previous works suggest that supplementation with exogenous digestive enzymes may hinder the development of metabolism in the digestive organs. Excessive levels of enzymes can affect levels of endogenous enzymes in the gastrointestinal tract, with negative effects on health[19]. Variations in the level of this effect depend on many factors, such as age, type of diet and enzyme dose[20–22]. We added NSP enzyme with different levels of proteases to the broiler diet to evaluate their effects on growth, physiological index, and the synthesis and secretion of endogenous enzymes. These findings provide evidence for the application of enzymes in poultry feed.

## Materials and methods

The research was conducted in accordance with the Declaration of Helsinki and with the Guide for Care and Use of Laboratory Animals as adopted and promulgated by the United National Institutes of Health. All experimental protocols were approved by the Review Committee for the Use of Institutional Animal Care and Use Committee of Henan Agricultural University. Institutional Animal Care and Use Committee of Henan Agricultural University approval Number (20161030).

### Chickens and diets

Three hundred one-day-old male broiler chicks (Ross-308) were randomly allocated by body weight to five treatments, with six replicate pens of 10 broilers. The broilers were housed in electrically heated cages with 24 h light and provided with food and water for 42 days. The room temperature was maintained at 33 to 35°C during the first week and was gradually decreased to 24°C by the end of the third week. The birds were fed a corn-soybean basal diet formulated by the National Research Council (Table 1). Additionally, 150 mg/kg NSP enzyme and 40, 80 or 160 mg/kg acid protease were mixed into the corn-soybean diet to produce the experimental diets. NSP enzyme (containing 20 000 U/g xylanase, 2500 U/g β-glucanase and 500 U/g cellulaseenzymatic activity) and acid protease (enzymatic activity 50 000 U/g) were obtained from the Shanghai Honest Biological Technology Co. Ltd. All diets were fed in mash form, The diets and water were supplied for *ad libitum* intake.\

**Table 1:** Diet compositions and nutrient levels.

Item	1-21 d	22-42 d
Ingredients, %		
Corn	51.31	54.60
Soybean meal	40.00	36.20
Soybean oil	4.60	5.60
Dicalcium phosphate	1.90	1.60
Limestone	1.30	1.20
NaCl	0.30	0.30
DL- Methionine	0.20	0.11
Choline chloride	0.20	0.20
Mineral premix^1^	0.10	0.10
Maduramicin Ammonium	0.06	0.06
Vitamin premix^2^	0.03	0.03
Total	100.00	100.00
Nutrient levels		
ME, kcal/kg	2984.72	3092.08
Crude protein	21.67	20.38
Met, %	0.55	0.44
Lys, %	1.10	1.02
Thr, %	0.91	0.84
Cys, %	0.37	0.35
Trp, %	0.31	0.28
Calcium, %	0.99	0.88
Total phosphorus, %	0.70	0.63
Non-phytate phosphorus, %	0.44	0.39

^1^Provided per kg of diet: vitamin A, 15000 IU; vitamin D_3_, 3900 IU; vitamin E, 30 IU; VK_3_, 3 mg; VB_1_, 2.4 mg; VB_2_ 9 mg; B_6_, 4.5 mg; B_12_, 0.021 mg; Pantothenic acid, 30 mg; Niacin, 45 mg; Folic acid, 1.2 mg; Biotin, 0.18 mg.

^2^Provided per kg of diet: Cu(CuSO_4_•5H_2_O), 8 mg; Zn(ZnSO_4_•7H_2_O), 40 mg; Fe(FeSO_4_•7H_2_O), 80 mg; Mn(MnSO_4_.5H_2_O), 100mg; I (KI), 0.35 mg; Se(Na_2_SeO_3_), 0.15 mg.

### Sample collection

The broilers were weighed after being starved for 12 h at 21 and 42 days of age, and feed consumption for each pen was recorded for the 1–21-day or 1–42-day phases. The average daily feed intake (ADFI), average daily gain (ADG), and feed-to-gain ratio (FGR) were recorded during the initial (1–21 days) and total (1–42 days) phases of the experiment.

From 14-16 d and 35-37 d, fecal samples were collected in duplicate and then dried at 60°C. Feed and stool samples were used to determine the apparent digestibility of crude protein (ADCP). At 21 and 42 d of age, one chick per cage was selected and weighed. The blood samples were taken from the wing vein, centrifuged at 2,500×g for 0.5 h at 4°C to separate the serum and then stored at -20°C until further analysis. The chickens were anesthetized and then killed by jugular bleeding. The pancreatic and intestinal contents were immediately frozen in liquid nitrogen and then stored at -70°C.

### Digestibility assays

Fecal and feed samples were dried at 105°C for 24 h and filtered through a 0.5-mm sieve. All samples were analyzed in duplicate. CP was determined using the AOAC method (2000)[23]. Acid-insoluble ash (AIA) was determined using the method of Choct and Annison (1992)[3]. Nutrient digestibility was calculated as follows:

Digestibility(%)=100−100×(dietAIA%÷fecalAIA%)×(fecalCP%÷dietCP%)

### Determination of cholecystokinin (CCK) in serum

Serum CCK was analyzed by ELISA using commercial kits (Kit number EK-069-04; PhoenixBiotech Co., LTD, Beijing, China).

### Determination of endogenous digestive enzyme activity in the pancreas

Approximately 1 g of pancreatic tissue was added to 9 ml distilled water to obtain a 100 mg·g^-1^ homogenate with Omni Prep Multi-Sample Homegenizer (Omni, USA), and then centrifuged at 1,500×g for 15 min. The supernatant was collected for the determination of digestive enzyme activities. Trypsin, lipase and amylase activity were determined using different kits (A080, A054 and C016, Nanjing Jiancheng Bioengineering Institute). Trypsin activity was defined as the amount of enzyme in each milligram of protein that caused an increase in absorbance of 0.003 per min at 253 nm wavelength, pH 8.0 and 37°C[24]. Lipase activity was determined per the amount of enzyme that hydrolyzed olive oil to form 1 μmol product per minute[25]. Amylase activity was defined as the amount of enzyme that hydrolyzed 1.0 mg substrate per 3 min at pH 6.9 at 40°C[26]. The protein concentrations were measured using the Coomassie Brilliant Blue method with bovine serum albumin as standard.

### Determination of the mRNA expression of pancreatic digestive-enzyme genes

#### RNA extraction

We extracted total RNA from approximately 50 mg of pancreatic tissue using TRIzol reagent according to the manufacturer’s instructions (Invitrogen, USA). The RNA concentration was determined by absorbance at 260 nm using a UV spectrophotometer (UV754N, Shenzhen, China). We evaluated RNA purity by measuring the OD260/OD280 ratio. The RNA samples typically had an OD260/OD280 ratio between 1.9 and 2.0.

#### Primer synthesis

The primers were synthesized by the Shanghai Biological Technology Co. (China). The pancreatic trypsin, amylase and lipase primer pairs (P1 and P2, P3 and P4, and P5 and P6) were designed based on the trypsin, amylase and lipase coding sequences, respectively. The β-actin primer pair (P7 and P8) was designed based on the conserved β-actin sequence. The trypsin, amylase, lipase and β-actin cDNA primers were designed based on the GenBank sequences NM_205385, NM_001001473, DQ 334850 and NM_205518, respectively (Table 2).

**Table 2:** Primer pairs for trypsin, amylase, lipase and β-actin genes from broilers.

Primers	Sequence	Length of fragment (bp)
Trypsin	P1 5’-AGCGAGCAGACCATTAGTTC-3’	253
	P2 5’-AGGAGAGTACAGGGGCATTC-3’	
Amylase	P3 5’-AAGTGGAATGGAGAGAAGATG-3’	147
	P4 5’- CCAGAAAGTAAGAATGGAAGC-3’	
Lipase	P5 5’-CCCTGCCCAACCTTATTT-3’	176
	P6 5’-GCATTTCCACTCCTCCGT-3’	
β-actin	P7 5’-ACCGCAAATGCTTCTAAAC-3’	93
	P8 5’-CCAATCTCGTCTTGTTTTATG-3’	

#### Fluorescence quantitative reverse transcription polymerase chain reaction (qRT-PCR)

We carried out a real-time PCR using a SYBR^®^ PrimeScript^™^ RT-PCR Kit (Takara Biotechnology, Dalian, China) according to the manufacturer’s instructions. Total RNA was reverse-transcribed to obtain cDNA using oligo-dT and reverse transcriptase at 50°C for 30 min and then heat inactivation of the enzyme at 85°C for 5 min. The cDNA samples were then mixed with SYBR^®^ dye. We carried out gene-specific and quantitative real-time PCR using a Bio-Rad iQ5 PCR machine (Bio-Rad, USA). The PCR program consisted of pre-denaturation at 95°C for 5 min, 35 cycles of denaturation at 95°C for 10 s, annealing at 55°C or 56°C (for β-actin and amylase, respectively) for 15 s and extension at 72°C for 12 s.The PCR was then heated from 60 to 95°C to produce the melting curve. One negative control was included in all reactions.

#### Digestive-enzyme mRNA

The quantity of digestive enzyme mRNA in each sample was normalized to β-actin. The cDNA of the digestive enzymes was quantified using relative standard-curve methods. Because the amplification efficiencies of the target and references genes were slightly different, the quantification of the gene copy number was obtained from different standard curves for the target and reference genes. The average value obtained for the control sample was defined as 1, and the experimental results are expressed as a percentage of those obtained for the control group.

https://www.ncbi.nlm.nih.gov/pubmed/11846609?dopt=Abstract

### Statistical analysis

The experimental data are expressed as means with standard errors. The data were analyzed using analysis of variance in SAS 8.0, and Duncan’s multiple-range test was used to compare treatment means. Differences were considered statistically significant at P < 0.05.

## Results

### Performance

The growth-performance results are presented in Table 3. For days 1-21, supplementary protease and NSP enzyme had no effect on average daily feed intake (ADFI) and average daily gain (ADG) (p>0.05), although the feed conversion ratio (FCR) was significantly improved with supplementation with NSP enzyme or NSP enzyme combined with 160 mg/kg protease compared to the control (p<0.05). For days 1-42, ADFI and ADG were significantly enhanced by NSP enzyme combined with protease at 40 or 80 mg/kg (p<0.05), and supplementation with NSP enzyme combined with protease at 80 mg/kg yielded the highest ADG (6.79% increase compared to the control) compared to the control diet. FCR was significantly improved compared to the control by supplementation with NSP enzyme or NSP enzyme combined with 40 or 80 mg/kg protease (p<0.05), but supplementation with NSP enzyme combined with 160 mg/kg protease had no effect on FCR (p>0.05).

**Table 3:** Effect of different levels of complex enzymes on body weight gain, feed intake, and feed conversion in broilers.

Item	Supplementary enzyme level (NSP enzyme + protease) (mg/kg)				
	1	2	3	4	5
	(0+0)	(150+0)	(150+40)	(150+80)	(150+160)
1–21 days					
ADFI	44.31 ± 1.05	44.04 ± 2.08	44.29 ± 2.65	45.81 ± 1.97	46.59 ± 0.91
ADG	28.51 ± 0.75	28.73 ± 1.50	28.74 ± 1.96	29.79 ± 1.35	30.44 ± 0.64
FCR	1.55 ± 0.01 ^a^	1.53 ± 0.01 ^b^	1.54 ± 0.02 ^ab^	1.54 ± 0.01 ^ab^	1.53 ± 0.01 ^b^
1–42 days					
ADFI	81.60 ± 1.42^cd^	80.95 ± 1.49^d^	84.16 ± 1.06^b^	86.26 ± 1.35^a^	83.37 ± 2.48^bc^
ADG	42.68 ± 0.70^b^	43.03 ± 0.90^b^	44.78 ± 0.72^a^	45.55 ± 0.59^a^	43.66 ± 1.32^b^
FCR	1.91 ± 0.01^a^	1.88 ± 0.01^b^	1.88 ± 0.01^b^	1.89 ± 0.02^b^	1.91 ± 0.01^a^

Each value represents mean ± SE of six replicates. In the same row, values with no superscript letter or the same superscript letter indicate no significant difference (P > 0.05); those with different superscript letters indicate a significant difference (P < 0.05). ADFI, average daily feed intake; ADG, average daily gain; FCR, feed conversion ratio.

### Digestibility

The results for the apparent digestibility of crude protein are presented in Table 4. For days 14-16, apparent digestibility of crude protein (ADCP) was significantly enhanced by supplementation with NSP enzyme or NSP enzyme combined with 40 or 160 mg/kg protease (p<0.05), and supplementation with NSP enzyme combined with protease at 160 mg/kg yielded the highest ADCP (7.26% increase compared to the control). For days 35-37, compared to the control, ADCP was significantly enhanced by supplementation with NSP enzyme or NSP enzyme combined with protease at 40 or 80 mg/kg (p<0.05), and supplementation with NSP enzyme combined with 40 mg/kg protease yielded the highest ADCP (12.66% increase compared to the control).

**Table 4:** Effect of different levels of complex enzymes on apparent digestibility of crude protein and serum CCK of broilers.

Item	Supplementary enzyme level (NSP enzyme + protease) (mg/kg)				
	1	2	3	4	5
	(0+0)	(150+0)	(150+40)	(150+80)	(150+160)
ADCP(%)					
14–16 days	65.14±1.40^c^	67.66±1.55^b^	67.10±1.49^b^	66.79±1.49^bc^	69.88±1.53^a^
35–37 days	54.90±1.06^c^	60.43±1.63^ab^	61.86±2.10^a^	59.40±1.32^b^	55.23±1.08^c^
CCK(pg/ml)					
21 days	98.22±41.40 ^a^	162.28±14.41 ^a^	134.38±36.80 ^a^	130.39±79.57 ^a^	127.93±48.62 ^a^
42 days	248.43±43.34 ^a^	314.30±11.82 ^a^	299.81±102.02 ^a^	283.72±58.47 ^a^	170.46±61.57 ^b^

Each value represents mean ± SE of six replicates. In the same row, values with no superscript letter or the same superscript letter indicate no significant difference (P > 0.05); those with different superscript letters indicate a significant difference (P < 0.05).

### Serum CCK

Results for the CCK level in the serum are shown in Table 4. At 21 days of age, compared to the control animals, the serum CCK in animals given NSP enzyme or NSP enzyme combined with protease supplementation at 40, 80 or 160 mg/kg (p>0.05). At 42 days of age, compared to the control animals, the CCK level in the serum of animals treated with NSP enzyme and protease at 160 mg/kg was reduced by 31.39% (p<0.05), but the CCK level in the serum of animals given NSP enzyme alone was increased by 26.51%.

### Activity of pancreatic digestive enzymes

The results for trypsin activity in the pancreas are presented in Table 5. After 21 days, compared to the control animals, animals given NSP enzyme or NSP enzyme combined with protease at 40 or 80 mg/kg showed increased activity of pancreatic trypsin (74.13%, 70.66% and 42.59%) (p<0.05), respectively. However, trypsin activity in the pancreas of the animals given NSP enzyme combined with 160 mg/kg protease group (245 U·mg protein^-1^) was similar to that observed in the control group (252 U·mg protein^-1^). After 42 days, supplementation with NSP enzyme or NSP enzyme combined with 40 mg/kg protease significantly increased the activity of pancreatic trypsin (32.45% and 27.41%) (p<0.05), compared with the control group. However, supplementation with NSP enzyme combined with protease at 80 or 160 mg/kg decreased the activity of pancreatic trypsin by 10.75% and 25.88% (p<0.05).

**Table 5:** Effect of different levels of complex enzymes on digestive enzyme activity of broilers pancreas (U/mgprot).

Item	Supplementary enzyme level (NSP enzyme + protease) (mg/kg)				
	1	2	3	4	5
	(0+0)	(150+0)	(150+40)	(150+80)	(150+160)
Trypsin					
21 days	251.94±28.20 ^c^	438.72±62.39 ^a^	429.97±20.77 ^a^	359.24±38.11 ^b^	244.87±31.58 ^c^
42 days	406.14±28.48 ^b^	537.95±46.30 ^a^	517.47±37.09 ^a^	362.50±21.22 ^c^	301.04±19.32 ^d^
Lipase					
21 days	475.43±14.70 ^c^	648.16±10.68 ^a^	601.47±19.96 ^b^	616.09±16.37 ^b^	606.53±22.26 ^b^
42 days	499.37±20.16 ^d^	784.36±32.78 ^b^	798.87±26.26 ^b^	892.81±18.38 ^a^	635.56±12.66 ^c^
Amylase					
21 days	117.69±6.39 ^b^	135.45±18.09 ^a^	133.91±1.68 ^a^	139.63±3.09 ^a^	129.59±18.93 ^ab^
42 days	178.83±3.75 ^e^	205.08±1.45 ^a^	201.26±2.07 ^a^	193.04±1.72 ^b^	195.63±6.17 ^b^

Each value represents mean ± SE of six replicates. In the same row, values with no superscript letter or the same superscript letter indicate no significant difference (P > 0.05); those with different superscript letters indicate a significant difference (P < 0.05).

Pancreatic lipase activity was significantly higher in treated groups than in the control group (p<0.05). Supplementation with NSP enzyme resulted in the highest levels of pancreatic lipase at 21 days of age (36.33% increase compared to the control group). After 42 days, supplementation with NSP enzyme combined with protease at 80 mg/kg had the highest pancreatic lipase (increased by 78.79% compared to the control group).

After 21 days, compared to the control, supplementation with NSP enzyme or NSP enzyme combined with protease at 40 or 80 mg/kg increased the activity of pancreatic amylase by 15.09%, 13.78% and 18.64% (p<0.05), respectively. After 42 days, pancreatic amylase activity was significantly higher in treated animals than in the control group (p<0.05).

### Expression of pancreatic digestive-enzyme mRNA

NSP enzyme or NSP enzyme combined with protease significantly affected trypsin, lipase and amylase mRNA expression in broilers (Table 6). After 21 days, trypsin mRNA expression in the pancreas had increased by 53%, 55%, 51% and 34% (P<0.05) in the animals given NSP enzyme and NSP enzyme combined with 40, 80 or 160 mg/kg protease, respectively. Compared to the control, after 42 days, animals given NSP enzyme, NSP enzyme combined with 40 or 80 mg/kg protease showed increased pancreatic trypsin mRNA levels (increased by 40%, 44% and 28%, respectively). However, supplementation with NSP enzyme combined with 160 mg/kg significantly decreased (13% difference) pancreatic trypsin mRNA levels. The broilers pancreatic lipase and amylase mRNA expression were significantly higher than those of control group (p<0.05).

**Table 6:** Effect of different levels of complex enzymes on Quantification of pancreatic digestive enzyme mRNA in broilers (relative ratio).

Item	Supplementary enzyme level (NSP enzyme + protease) (mg/kg)				
	1	2	3	4	5
	(0+0)	(150+0)	(150+40)	(150+80)	(150+160)
Trypsin					
21 days	1.00±0.09 ^c^	1.53±0.10 ^a^	1.55±0.10 ^a^	1.51±0.08 ^a^	1.35±0.06 ^b^
42 days	1.00±0.12 ^c^	1.40±0.06 ^a^	1.44±0.08 ^a^	1.28±0.03 ^b^	0.87±0.11 ^d^
Lipase					
21 days	1.00±0.07 ^b^	1.27±0.07 ^a^	1.24±0.02 ^a^	1.26±0.01 ^a^	1.23±0.03 ^a^
42 days	1.00±0.07 ^d^	1.15±0.02 ^a^	1.12±0.03 ^ab^	1.13±0.04 ^ab^	1.07±0.07 ^bc^
Amylase					
21 days	1.00±0.05 ^b^	1.79±0.04 ^a^	1.77±0.04 ^a^	1.78±0.02 ^a^	1.80±0.06 ^a^
42 days	1.00±0.04 ^b^	1.61±0.06 ^a^	1.56±0.04 ^a^	1.60±0.04 ^a^	1.59±0.03 ^a^

Each value represents mean ± SE of six replicates. In the same row, values with no superscript letter or the same superscript letter indicate no significant difference (P > 0.05); those with different superscript letters indicate a significant difference (P < 0.05).

## Discussion

Amino acids are vital nutrients derived from food proteins (except for synthetic forms). Protein degradation is largely determined by the secretion of the pancreas and stomach. Protein digestibility in broiler diets changes with dietary composition[27, 28]. Thus, incomplete digestion, including undigested proteins,can be ameliorated by the addition of exogenous proteases. Arabinoxylan is an important water-soluble non-starch polysaccharide (NSP) in feed that increases chyme viscosity, decreases the availability of nutrients, and alters the intestinal flora, thus affecting nutrient digestion and absorption[3, 29]. Previous studies have shown that the addition of xylanase to wheat-based diets significantly increases body weight gain (BWG) and improves the feed conversion rate (FCR) of broilers at 21 days of age[14, 30, 31] without affecting feed intake, indicating that improved nutrient utilization was responsible for the improved feed-conversion rate[32]. In this study, at 1-21 days and 1-42 days, the FCR was significantly improved compared to the control by supplementation with NSP enzyme alone, but there was no significant effect on ADG and ADFI. The effects of exogenous proteases added to poultry diets on growth performance are variable, possibly due to differences in tests and trial designs, especially in the negative control diets. Additionally, many studies have used complexes of enzymes rather than a single component enzyme, yielding more varied results[1, 10, 15, 33–36] because the results cannot be attributed to one particular enzyme. The present study demonstrated that during days1-42, the addition of NSP enzyme combined with a protease at 40 or 80 mg/kg to a corn-soybean meal diet can increase ADFI and ADG.

The results showed that addition of this enzyme could improve the FCR and ADG of broilers by increasing protein digestibility. At 21 days of age, CP was significantly enhanced by supplementation with NSP enzyme or NSP enzyme combined with 40 or 160 mg/kg protease (p<0.05). At 42 days of age, compared to the control, CP was significantly enhanced by supplementation with NSP enzyme or NSP enzyme combined with the protease at 40 or 80 mg/kg (p<0.05). Previous research results show that the addition of xylanase to wheat-based diets significantly reduces the viscosity of the digesta and improves the absorption of DM, CP and calories in broilers, consistent with our results[31]. Corn-soybean-meal diets supplemented with compound enzyme (containing xylanase, protease and amylase) could improve crude protein digestibility by 15.6% while increasing body weight gain by 5.5% and FE by 4%[35]. The same enzyme used in corn-soybean-meal and wheat middlings-based diets increased crude-protein availability by 26.3% but had no effect on growth performance[37]. Therefore, our findings, along with those of Cowieson et al. [35] and Freitas et al. [38], suggest that multienzyme complexes can improve nutrient availability and protein digestibility.

The intestinal tract has endocrine cells that secrete CCK, and 98% of the CCK in the intestinal tract is present in the mucosal layer, with the highest concentration in the duodenum. CCK has an important physiological function in the gastro intestinal tract, regulating pancreatic exocrine secretion and promoting the synthesis of trypsin, chymotrypsin and amylase. CCK also inhibits gastric emptying, promotes bowel movements, and causes gallbladder contraction[39]. CCK A Receptor (CCK-_A_R) is mainly distributed in the pancreatic gland, gallbladder, smooth muscle, vagus-nerve afferent fibers, brain etc.[40]. CCK-_A_R in gastrointestinal tract is mainly mediated by secretion from the pancreas, contraction of the gallbladder and gastric smooth muscle and secretion from intestinal mucosal cells. It enhances sphincter muscle tension to delay gastric emptying[41] and reduces feed intake[42, 43]. Feed intake can be increased by reducing levels of CCK[44]. In this study, the level of CCK in serum was increased with NSP enzyme supplementation. However, with increased protease levels, CCK levels gradually decreased, and the ADFI increased significantly during days1-42. This research indicated that exogenous protease could increase the feed intake of broilers by decreasing serum levels of CCK.

The amount of digestive juice synthesized or secreted by the pancreas, liver and intestinal mucosa and the increased enzymatic activity determine digestive function. Pancreatic juice includes a variety of enzymes involved in the degradation of nutrients, such as pancreatic amylase, trypsin, chymotrypsin, elastase, pancreatic lipase and colipase, all of which are directly related to digestive function. The strength of pancreatic enzyme activity is an excellent index by which to measure the digestive capacity[17]. In the present study, supplementation with NSP enzyme or NSP enzyme combined with protease significantly increased the activity of pancreatic lipase and amylase in broilers, but supplementation with NSP enzyme combined with protease at 80 or 160 mg/kg significantly decreased the activity of pancreatic trypsin. Almirall et al. reported that supplementary β-glucanase enzyme significantly increased the activity of lipase, amylase and trypsin in the intestinal contents of broilers on a barley-based diet[45]. Engberg et al. also found that supplementary xylanase significantly increased the activity of lipase and chymotrypsin in broilers[46]. A rat experiment showed that pancreatic secretion and enzyme activity increased significantly as active polysaccharide was added[47]. Mahagna et al. reported that supplementary amylase and protease significantly decreased the activity of amylase, chymotrypsin and trypsin in the intestinal contents of broilers on asorghum-soybean-meal diet[48]. Cowieson et al. added glucose solution containing seven types of monomeric enzymes to broiler diets and found that both phytase and protease significantly increased the excretion of endogenous nitrogen, amino acids, calories and dry matter, suggesting that exogenous proteases may participate in the feedback regulation of the pancreas[19]. Therefore, supplementation with exogenous digestive enzymes would decrease the activity of pancreatic digestive enzymes, and supplementation of viscous grain diets with NSP enzyme could reduce the inhibitory effect of NSP on digestive-enzyme activity.

The synthesis of pancreatic digestive enzyme is controlled by a specific gene in acinar cells. The enzyme precursors are modified into zymogens, which are then transported to the top of the cell by the Golgi apparatus and packaged into vacuoles. In response to an external signal, the cell secretes the zymogen into the pancreatic duct[49]. In the present study, after 42 days, supplementation with NSP enzyme or NSP enzyme combined with 40 or 80 mg/kg protease significantly increased levels of pancreatic trypsin, lipase and amylase mRNA, but supplementation with NSP enzyme combined with 160 mg/kg of protease significantly decreased pancreatic trypsin mRNA. Endogenous expression of digestive enzyme genesis regulated by dietary nutrient levels. A study on rats showed that when dietary protein content increased from 15% to 70%, pancreatic trypsin, chymotrypsin, and elastase expression increased 3.6, 3.9 and 1.8 times, respectively[50]. When the carbohydrate content increased from 11% to 75%, pancreatic amylase mRNA increased 3.5-8 times[51]. It has been reported that when dietary fat content increased from 3% to 30%, lipase expression increased 2.2-3.9 times[52]. Thus, endogenous expression of digestive-enzyme genes may be regulated through nutrition.

## Conclusions

Supplementation with NSP enzyme and protease at 80 mg/kg yielded the best performance in ADG broilers.The addition of 150 mg/kg NSP enzyme improved FCR, promoted serum CCK secretion, increased the expression of pancreatic trypsin, amylase and lipase genes, and enhanced digestive enzyme activity in the pancreas. When the NSP enzyme was combined with 160 mg/kg protease, the growth of broilers was significantly improved during days 1-21 but decreased in the later stage. Broiler serum CCK, pancreatic trypsin activity and mRNA expression were decreased with the increase of exogenous protease, and the inhibitory effect was more obvious in the later stage.

## Supporting information

S1 File Effect of different levels of complex enzymes on body weight gain, feed intake, and feed conversion in broilers. (XLS)

S2 File Effect of different levels of complex enzymes on apparent digestibility of crude protein and serum CCK of broilers. (XLS)

S3 File Effect of different levels of complex enzymes on digestive enzyme activity of broilers pancreas (U/mgprot). (XLS)

S4 File Effect of different levels of complex enzymes on Quantification of pancreatic digestive enzyme mRNA in broilers (relative ratio). (XLS)
